# Distinguishing feature of gut microbiota in Tibetan highland coronary artery disease patients and its link with diet

**DOI:** 10.1038/s41598-021-98075-9

**Published:** 2021-09-16

**Authors:** Yulan Ma, Lulu Zhu, Zhijun Ma, Zhongshan Gao, Yumiao Wei, Youlu Shen, Lin Li, Xingli Liu, Ming Ren

**Affiliations:** 1 Department of Cardiology, Affiliated Hospital of Qinghai University, Xining, 810001 China; 2 Department of Surgical Oncology, Affiliated Hospital of Qinghai University, Xining, 810001 China; 3 Laboratory of Cardiovascular Immunology, Institute of Cardiology, Union Hospital, Tongji Medical College, Huazhong University of Science & Technology, Wuhan, China

**Keywords:** Unstable angina, Clinical microbiology, Microbial communities

## Abstract

The prevalence of coronary artery disease (CAD) in Tibetan Highlanders is lower than that in plain-living individuals, but the mechanism still unclear. Gut microbiota (GM) disorder is considered one of the potential factors involved in the pathogenesis of CAD, but the GM characteristics of Tibetan Highlanders suffering from CAD are unknown. We sequenced the V3-V4 region of the 16S ribosomal RNA of gut bacteria from fecal samples from Tibetan and Han CAD patients and healthy individuals inhabiting the Qinghai-Tibet Plateau, as well as from Han CAD patients and healthy individuals living at sea level, and we analyzed the GM characteristics of these subjects by bioinformatics analysis. The results showed that Tibetan Highlanders suffering from CAD had higher GM α-diversity, with differently distributed cluster compared with healthy Tibetan Highlanders and Han CAD patients living at high and low altitudes. Genera *Catenibacterium, Clostridium_sensu_stricto*, *Holdemanella*, and *Ruminococcus 2* were enriched in Tibetan Highlanders suffering from CAD compared with healthy Tibetan Highlanders and Han CAD patients living at high- and low-altitudes. *Prevotella* was enriched in Tibetan Highlanders suffering from CAD compared with Han CAD patients living at high- and low-altitudes. Moreover, *Catenibacterium* was positively correlated with *Prevotella*. Additionally, *Catenibacterium, Holdemanella*, and *Prevotella* were positively correlated with fermented dairy product, carbohydrate and fiber intake by the subjects, while *Clostridium_sensu_stricto* was negatively correlated with protein intake by the subjects. In conclusion, our study indicated that Tibetan Highlanders suffering from CAD showed distinct GM, which was linked to their unique dietary characteristics and might associated with CAD.

## Introduction

Coronary artery disease (CAD) is one of the most harmful diseases that threatens human health. Because of hypobaric hypoxia, high altitude, low temperature, and high ultraviolet rays, plateau area in the world, especially the Qinghai-Tibet Plateau (QTP), are harsh environments for human survival. Epidemiological surveys have demonstrated that most chronic diseases are prevalent in these regions, such as hypertension and chronic kidney disease^1–3^. However, Fujimoto et al.^4^ reported that Tibetan Highlanders showed a lower incidence of CAD than plain-living Japanese. Mortimer et al.^5^ found a negative correlation between CAD mortality and living altitude in male New Mexicans. A study in Switzerland also confirmed lower CAD mortality at higher altitudes^6^. Anatomically, it was found that high-altitude rodents showed a higher density of collaterals than lowland rodents^7^. Therefore, high-altitude living could be beneficial for the progression of CAD, but the mechanism remains unclear.

In recent years, the gut microbiota (GM) has been increasingly understood. Dysbiosis of the GM is closely related to cardiovascular diseases, including CAD. Studies have confirmed that GM disorder is involved in the development of atherosclerosis and the formation of CAD^8^. Many studies have shown that there were significant differences in the structure and composition of intestinal flora between the healthy groups and the CAD groups^9,10^. In animal experiments, Liu et al.^11^ found an abnormal GM structure, elevated blood cholesterol, imbalanced helper T cells proportions, and enhanced secretion of inflammatory cytokines in mice colonized with feces from CAD patients. Further studies demonstrated that abundant of gut bacterial DNA was found in atherosclerotic lesions, which indicated that the GM might directly participate in the pathogenesis of CAD^12,13^. GM metabolites are also involved in the development of CAD. Trimethylamine N‐oxide (TMAO) can accelerate atherosclerosis by inhibiting reverse cholesterol transport and accumulating macrophage cholesterol^14^. Short-chain fatty acids (SCFAs) might decelerate atherosclerosis by inhibiting inflammation^15^.

In addition to host health conditions, the diversity and composition of the GM are influenced by other factors, including host genetic background, dietary habits and environmental factors. Li et al.^16^ found that the GM of plain-living Han individuals was significantly different from that of plateau-living Tibetan individuals. Jia et al.^17^ confirmed that the GM of Han individuals changed to adapt to the plateau environment after entering the plateau and became increasingly similar to the GM of the Tibetan individuals. Therefore, we supposed that the GM of Tibetan Highlanders suffering from CAD may have specific characteristics different from those of other populations, which has still not been shown in any other studies.

In this study, we recruited QTP-living Tibetan and Han CAD patients (HTC and HHC, respectively), healthy QTP-living Tibetan and Han individuals (HTN and HHN, respectively), and plain-living Han CAD patients and healthy plain-living Han individuals (LHC and LHN, respectively). By analyzing the GM of these subjects, we explored the GM characteristics of Tibetan Highlanders suffering from CAD and highlighted the relationship between diet structure and specific genera.

## Results

### Participant general characteristics and diet structure

Age, sex, body mass index (BMI), smoking status, systolic blood pressure (SBP) and diastolic blood pressure (DBP) on admission, total cholesterol (TC), triglycerides (TG), low-density lipoproteins (LDL-c), alanine aminotransferase (ALT), aspartate aminotransferase (AST), urea nitrogen (BUN), creatinine, fasting blood glucose, and c-reactive protein (CRP) levels were noted for each participant. Cardiac function and geometry, including left atrium diameter (LA), right atrium diameter (RA), interventricular septal thickness (IVS), left ventricular end-diastolic diameter (LVEDD), right ventricle diameter (RV), and ejection fraction (EF%) were collected. For CHD patients, coronary anatomy and time since last chest pain were also collected. Age, sex, BMI, smoking status, SBP, DBP, TG, ALT, AST, BUN, creatinine, fasting blood glucose, CRP levels and cardiac function and geometry (including LA, RA, IVS, LVEDD, RV and EF%) showed no significant differences among the 6 groups (continuous variables: one-way analysis of variance (ANOVA), categorical variables: Kruskal–Wallis test, probability (*p*) > 0.05, Table 1). The No. of stenosed vessels and time since last chest pain also showed no significant differences within the CAD subgroups (continuous variables: ANOVA, categorical variables: Kruskal–Wallis test, *p* > 0.05, Table 1). The HHC group showed lower TC and LDL-c levels than the HHN group (two-tailed Student’s *t*-test, TC: *t* = 3.217, false discovery rate (FDR)-adjusted *p* = 0.009; LDL-c: *t* = 2.803, FDR-adjusted *p* = 0.063), the LHC groups showed lower TC levels than the HHN group (two-tailed Student’s *t*-test, *t* = 3.664, FDR-adjusted *p* = 0.009), which might attributed to statins use by CAD patients. We also analyzed the diet composition of subjects from different groups. Tibetan Highlanders consumed more fermented dairy products, carbohydrates and fiber but less protein and fat than high-altitude and low-altitude Han individuals (Kruskal–Wallis test, *p* < 0.05, Table 2).

**Table 1:** General clinical information of participants in different groups.

Participant characteristics	HTC (n = 8)	HTN (n = 34)	HHC (n = 14)	HHN (n = 35)	LHC (n = 14)	LHN (n = 35)	*p*	F/K
Age, years	57.6 ± 9.7	52.4 ± 6.5	58.4 ± 12.1	51.5 ± 7.3	54.9 ± 8.3	53.0 ± 6.8	0.053	2.251
Sex, male/%	5 / 62.5%	23 / 67.6%	10 / 62.5%	20 / 57.7%	10 / 71.4%	21 / 60.0%	0.909	1.532
BMI, Kg/m^2^	24.4 ± 1.9	23.7 ± 4.5	23.9 ± 1.2	23.5 ± 1.1	25.1 ± 2.1	24.0 ± 2.1	0.488	0.893
Smoking, n/%	1 / 12.5%	8 / 23.5%	8 / 57.1%	7 / 20.0%	5 / 35.7%	10 / 28.6%	0.121	8.723
SBP, mmHg	121.0 ± 12.1	119.2 ± 4.4	121.2 ± 6.3	118.0 ± 4.1	124.9 ± 14.2	120.3 ± 8.4	0.114	1.861
DBP, mmHg	75.1 ± 5.8	76.5 ± 6.3	78.6 ± 5.4	78.6 ± 3.6	75.6 ± 7.8	76.3 ± 7.5	0.367	1.093
TC, mmol/L	3.89 ± 0.88	4.23 ± 1.04	3.34 ± 0.49	4.36 ± 1.14	3.30 ± 0.89	4.13 ± 0.64	0.001	4.732
TG, mmol/L	1.92 ± 0.66	1.52 ± 0.73	1.58 ± 0.57	1.71 ± 0.97	1.54 ± 0.68	1.29 ± 0.53	0.161	1.613
LDL-c, mmol/L	2.10 ± 0.94	2.82 ± 0.94	1.97 ± 0.56	2.67 ± 0.87	1.84 ± 0.79	2.30 ± 0.80	0.001	4.658
ALT (U/I)	26.00 ± 7.80	25.79 ± 7.21	26.43 ± 7.23	28.14 ± 8.93	26.07 ± 8.77	27.06 ± 9.39	0.322	0.899
AST (U/I)	23.63 ± 5.40	24.12 ± 6.99	24.21 ± 6.70	24.11 ± 7.39	25.86 ± 9.25	24.40 ± 6.65	0.156	0.978
BUN (mmol/L)	4.35 ± 1.53	3.90 ± 0.97	4.53 ± 1.51	4.27 ± 1.12	4.28 ± 1.30	4.00 ± 1.17	0.841	0.523
Creatinine (umol/L)	68.13 ± 11.22	70.15 ± 13.34	69.29 ± 13.62	70.23 ± 10.93	70.21 ± 10.24	67.29 ± 10.66	0.318	0.901
Fasting blood glucose (mmol/L)	4.37 ± 0.62	4.41 ± 0.68	4.57 ± 0.81	4.48 ± 0.67	4.66 ± 0.51	4.62 ± 0.61	0.611	0.692
CRP (mg/L)	4.4 ± 1.4	4.6 ± 1.5	4.3 ± 1.1	4.9 ± 1.4	4.1 ± 1.1	4.9 ± 1.4	1.366	0.241
**Cardiac function and geometry**								
LA(mm)	34.4 ± 1.4	33.7 ± 3.2	33.3 ± 3.0	33.2 ± 3.1	35.9 ± 4.1	34.1 ± 3.4	1.575	0.171
LVEDD(mm)	45.1 ± 2.8	46.1 ± 5.0	46.4 ± 5.0	45.9 ± 4.3	46.6 ± 5.3	46.7 ± 5.0	0.202	0.961
IVS (mm)	7.4 ± 1.2	7.4 ± 0.9	7.3 ± 1.0	7.5 ± 1.0	7.6 ± 1.3	7.0 ± 1.0	1.117	0.354
RA(mm)	35.0 ± 3.5	34.2 ± 3.4	33.6 ± 3.6	34.5 ± 3.9	34.1 ± 2.9	34.4 ± 2.8	0.227	0.950
RV(mm)	22.9 ± 2.2	25.4 ± 4.5	23.1 ± 2.9	24.2 ± 3.8	25.0 ± 4.2	25.9 ± 4.6	1.616	0.160
EF(%)	59.3 ± 0.7	61.4 ± 5.9	63.1 ± 9.6	60.6 ± 5.7	61.8 ± 5.9	61.1 ± 5.1	0.570	0.723
**No. of stenosed vessels**								
Single-vessel, n / %	4 / 50.0%	6 / 42.9%	8 / 57.1%	NA	NA	NA	2.087	0.352
Double-vessel, n / %	1 / 12.5%	3 / 21.4%	4 / 28.6%	NA	NA	NA	0.769	0.681
Triple-vessel, n / %	3 / 37.5%	5 / 35.7%	2 / 14.3%	NA	NA	NA	0.488	0.893
Time since last chest pain (h)	3.6 ± 1.3	4.1 ± 1.4	4.1 ± 1.1	NA	NA	NA	0.381	0.686

For normally distributed measurement data and categorical variables, values are expressed as mean ± standard deviation (SD) and number / the percentage (n / %), respectively. *P* and F / K values are shown in the right column of the table, and *p* < 0.05 was considered statistically significant.

*CAD* coronary artery disease, *QTP* Qinghai-Tibet Plateau, *HTC* the Tibetan CAD patients residing at QTP, *HTN* the Tibetan healthy individuals residing at QTP, *HHC* the Han CAD patients residing at QTP, *HHN* the Han healthy individuals residing at QTP, *LHC* the Han CAD patients from sea level in southern China, *LHN* the Han healthy individuals from sea level in southern China, *BMI* body mass index, *SBP* systolic blood pressure, *DBP* diastolic blood pressure, *TC* total cholesterol, *TG* triglyceride, *LDL-c* low-density lipoprotein cholesterol, *ALT* alanine aminotransferase, *AST* aspartate aminotransferase, *BUN* urea nitrogen, *CRP* c-reactive protein, *LA* left atrium diameter, *RA* right atrium diameter, *IVS* interventricular septal thickness, *LVEDD* left ventricular end-diastolic diameter, *RV* right ventricle diameter, *EF%* ejection fraction, *NA* not available.

**Table 2:** Diet composition of participants in different groups.

Diet composition	HTC (n = 8)	HTN (n = 34)	HHC (n = 14)	HHN (n = 35)	LHC (n = 14)	LHN (n = 35)	*p*	K
Fermented products	35.0 (31.3, 40.0)	38.8 (34.4, 42.5)	17.5 (13.8, 25.0)	17.5 (10.0, 25.0)	12.5 (11.3, 15.0)	15.0 (10.0, 22.5)	0.000	73.414
Protein	75.0 (50.0, 100.0)	100.0 (50.0, 150.0)	100.0 (93.8, 150.0)	100.0 (100.0, 150.9)	100.0 (50.0, 125)	100.0 (50.0, 125)	0.002	19.121
Carbohydrates	350.0 (225.0, 387.5)	200.0 (165.0, 300.0)	200.0 (165.0, 262.5)	180.0 (150.0, 200.0)	200.0 (152.5, 262.5)	150.0 (140.0, 200.0)	0.001	20.901
Fat	45.0 (32.5, 50.0)	60.0 (50.0, 75.0)	60.0 (47.5, 66.3)	50.0 (30.0, 60.0)	60.0 (50.0, 70.0)	50.0 (30.0, 60.0)	0.004	17.207
Fiber	68.8 (50.0, 75.0)	50.0 (40.0, 61.7)	41.3 (32.5, 53.1)	37.5 (35.0, 50.0)	33.8 (30.0, 37.5)	40.0 (32.5, 50.0)	0.000	34.768

Non-normally distributed measurement data are expressed as median (25^th^ percentile, 75^th^ percentile). *P* and K values are shown in the right column of the table, and *p* < 0.05 was considered statistically significant.

*CAD* coronary artery disease, *QTP* Qinghai-Tibet Plateau, *HTC* the Tibetan CAD patients residing at QTP, *HTN* the Tibetan healthy individuals residing at QTP, *HHC* the Han CAD patients residing at QTP, *HHN* the Han healthy individuals residing at QTP, *LHC* the Han CAD patients from sea level in southern China; *LHN* the Han healthy individuals from sea level in southern China.

### Multivariable statistical analysis GM diversity and distribution among different groups

**Figure 1:**
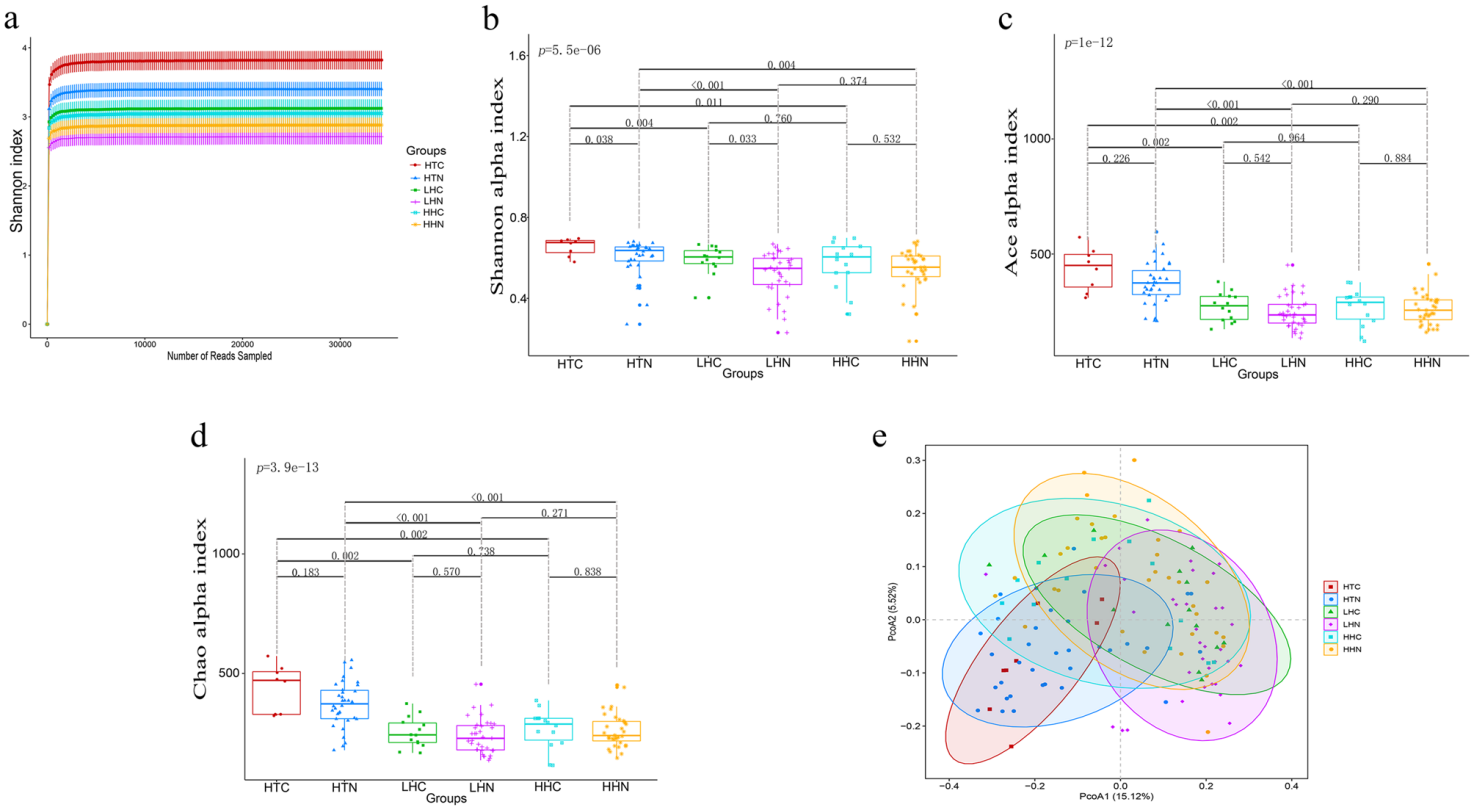
Shannon rarefaction curves, α-diversity, and β-diversity of gut microbiota among the groups. (**a**) Shannon rarefaction curves based on OTUs among different groups are shown as a similarity threshold of 97% . 95% confidence intervals are shown as error bars. Rarefaction curves of different groups are shown in different colors. (**b**–**d**) α diversity based on the Shannon (Mothur sofware v.1.43.0, http://www.mothur.org/wiki/Shannon) (**b**), Ace (Mothur sofware v.1.43.0, http://www.mothur.org/wiki/Ace) (**c**), and Chao (Mothur sofware v.1.43.0, http://www.mothur.org/wiki/Chao) (**d**) index in each group are shown in the boxplots, respectively. Different colored and shaped dots represent different groups of samples. The short horizontal lines of box from top to bottom stands for 75th, 50th, and 25th percentiles, respectively. Every two sub-groups are compared in pairs by two-tailed Student’s *t*-test and FDR adjusted *p-*values are attached in the horizontal lines. **e.** Weighted UniFrac distance matrices based PCoA is shown as bi-dimensional scatter plots of PCoA1 and PCoA2 axis (“vegan” package in R sofware v.3.6.0, https://www.r-project.org/). Different colored and shaped dots represent different groups of samples, and different colored ellipse represents 95% confidence intervals for each group. *CAD* coronary artery disease, *QTP* Qinghai-Tibet Plateau, *HTC* the Tibetan CAD patients residing at QTP, *HTN* the Tibetan healthy individuals residing at QTP, *HHC* the Han CAD patients residing at QTP, *HHN* the Han healthy individuals residing at QTP, *LHC* the Han CAD patients from sea level in southern China, *LHN* the Han healthy individuals from sea level in southern China; operational taxonomic units (OTUs); *PCoA* principal coordinate analysis.

First, the Shannon rarefaction curves based on the operational taxonomic units (OTUs) profiles for all samples reached a plateau, revealing that the sequencing depth sufficiently captures the GM variation in these populations (Fig. 1a). Second, to demonstrate the diversity of the GM, α-diversity analysis based on the Shannon, Ace, and Chao indexes were compared among the 6 groups. The multivariable analysis results showed that there were statistical differences in α-diversity among the 6 groups (one-way ANOVA, Shannon: *p* = 5.5e−06, Ace: *p* = 1e−12, Chao: *p* = 3.9e−13). The paired groups comparing analysis results showed that the Shannon index between HTC and HTN (two-tailed Student’s *t*-test, FDR-adjusted *p* = 0.038) and between LHC and LHN (two-tailed Student’s *t*-test, FDR-adjusted *p* = 0.033), showed significant differences (Fig. 1b–d). Among the CAD subgroups, the Shannon, Ace, and Chao indexes of the HTC subgroup were significantly higher than those of the HHC and LHC subgroups (two-tailed Student’s *t*-test, FDR-adjusted *p* < 0.005, Fig. 1b–d). Among the healthy individual subgroups, the Shannon, Ace, and Chao indexes of the HTN subgroup were significantly higher than those of the HHN and LHN subgroups (two-tailed Student’s *t*-test, FDR-adjusted *p* ≤ 0.005, Fig. 1b–d). However, no difference was shown between the HHC and LHC subgroups, and no difference was shown between the HHN and LHN subgroups (two-tailed Student’s *t*-test, FDR-adjusted *p* > 0.05, Fig. 1b–d). In conclusion, Tibetans living at high altitude showed higher α-diversity than Han individuals living at high- or low- altitude. Moreover, Tibetan CAD patients living at high altitude showed higher Shannon index than that of healthy Tibetan individuals living at high altitude. Furthermore, weighted UniFrac distance matrix-based principal coordinate analysis (PCoA) was used to estimate the β-diversity. In general, bacterial communities distributions of the 6 groups were different (adonis test, R^2^ = 0.106, *p* = 0.001, Table 2). Within the altitude- and ethnicity-matched subgroups, the bacterial community of HTC was different from HTN (adonis test: R^2^ = 0.034, FDR-adjusted *p* = 0.078), and the bacterial community of LHC was different from LHN (adonis test: R^2^ = 0.033, FDR-adjusted *p* = 0.057), but no significant difference was shown between HHC and HHN (adonis test: R^2^ = 0.016, FDR-adjusted *p* = 0.763, Fig. 1e and Table 3). Within the CAD subgroups, the bacterial community of HTC, HHC and LHC formed distinct clusters with each other (adonis test, HTC vs. HHC: R^2^ = 0.102 FDR-adjusted *p* = 0.002; HTC vs. LHC: R^2^ = 0.137, FDR-adjusted *p* = 0.002, HHC vs. LHC: R^2^ = 0.078, FDR-adjusted *p* = 0.005, Fig. 1e and Table 3). Within healthy individual subgroups, the bacterial community of HTC, HHC and LHC also formed distinct clusters with each other (adonis test, HTN vs. HHN: R^2^ = 0.038, FDR-adjusted *p* = 0.002; HTN vs. LHN: R^2^ = 0.088, FDR-adjusted *p* = 0.002, HHN vs. LHN: R^2^ = 0.076, FDR-adjusted *p* = 0.002, Fig. 1e and Table 3). In summary, GM distribution of Tibetan CAD patients living at high altitudes was slightly different from that of healthy Tibetan individuals living at high altitudes, but obviously different from that of Han CAD patients living at high or low altitude. And GM distribution of healthy Tibetan Highlanders also obviously different from that of healthy Hans living at high or low altitude. There results indicated that ethnicity, altitude, and CAD status were all contributors on the diversity and distribution of GM.

**Table 3:** Comparison microbiota composition among the groups by adonis test base on weighted UniFrac distance matrices.

Comparison	R^2^	FDR adjusted *p* Value / *p* value
HTC vs. HTN	0.034	0.078*
HHC vs. HHN	0.016	0.763
LHC vs. LHN	0.033	0.057*
HTC vs. HHC	0.102	0.002*
HTC vs. LHC	0.137	0.002*
LHC vs. HHC	0.078	0.005*
HHN vs. LHN	0.076	0.002*
HTN vs. LHN	0.088	0.002*
HTN vs. HHN	0.038	0.002*
Between	0.106	0.001^#^

R^2^ value is percent of explain dispersion. *p < 0.05 and FDR < 0.08; ^#^*p* < 0.05.

*CAD* coronary artery disease, *QTP* Qinghai-Tibet Plateau, *HTC* the Tibetan CAD patients residing at QTP, *HTN* the Tibetan healthy individuals residing at QTP, *HHC* the Han CAD patients residing at QTP, *HHN* the Han healthy individuals residing at QTP, *LHC* the Han CAD patients from sea level in southern China, *LHC* the Han healthy individuals from sea level in southern China.

### Different composition of GM among different groups

**Figure 2:**
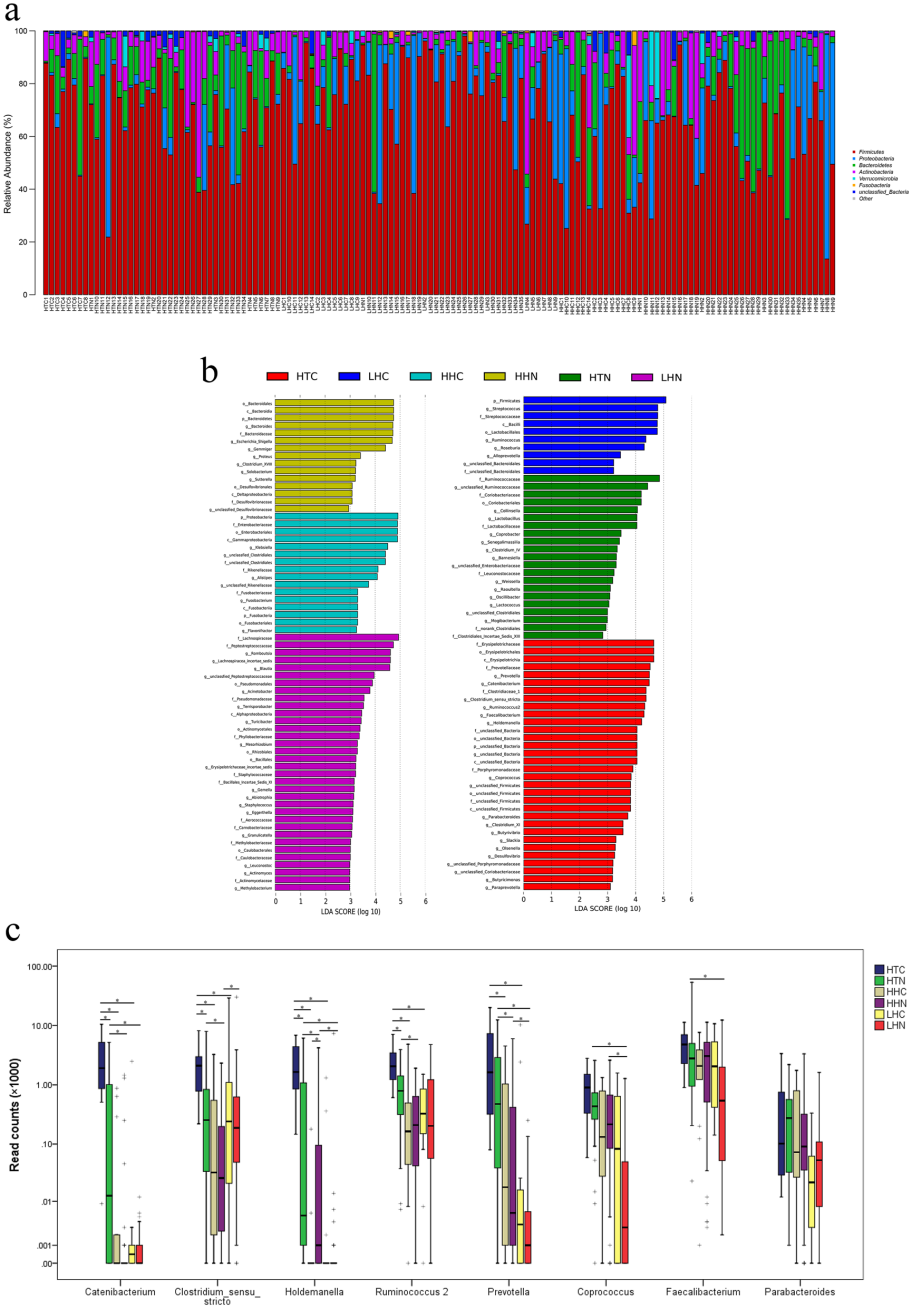
Taxonomic analysis of GM alterations in different groups. (**a**) The proportion of the first 5 dominant phyla of GM in each individual is displayed in the barplot and different phyla are shown in different colors (“circlize” package in R sofware v.3.6.0, https://www.r-project.org/). (**b**) LEfSe analysis are shown in the barplot (LEfSe sofware v.1.1.0, http://huttenhower.sph.harvard.edu/lefse/). Taxa enriched in different groups are indicated with different color. The horizontal axis is the LDA score, and the vertical axis is the group of microorganisms with significant effects. Only taxa meeting *p* < 0.05 and LDA > 2 are shown. Taxon abbreviations are added before the bacterium. *p* phylum, *c* class, *o* order, *f* family, *g* genus. (**c**) Cluster box plot compared the differences of the first 8 HTC-enriched genera (average read counts > 100/sample) at the read count level in every two groups in pairs by FDR-adjusted Wilcoxon rank sum test. *p < 0.05 and FDR < 0.05. *CAD* coronary artery disease, *GM* gut microbiota, *QTP* Qinghai-Tibet Plateau, *HTC* the Tibetan CAD patients residing at QTP, *HTN* the Tibetan healthy individuals residing at QTP; *HHC* the Han CAD patients residing at QTP, *HHN* the Han healthy individuals residing at QTP, *LHC* the Han CAD patients from sea level in southern China, *LHN* the Han healthy individuals from sea level in southern China, *LDA* Linear discriminant analysis, *LEfSe* LDA coupled with effect size.

Firstly, the abundance of the first 5 dominant phyla (Firmicutes, Proteobacteria, Bacteroidetes, Actinobacteria, and Verrucomicrobia) in the 6 groups is illustrated. We found that the distribution of the dominant phyla showed no statistical significance (one-way ANOVA, *p* > 0.05, Fig. 2a). Secondly, the specific bacteria in different groups were determined by Linear discriminant analysis (LDA) coupled with effect size (LEfSe). 15 genera were significantly enriched in the HTC group: *Prevotella, Catenibacterium, Clostridium_sensu_stricto, Ruminococcus 2, Faecalibacterium, Holdemanella, Coprococcus, Parabacteroides, Clostridium_XI, Butyrivibrio, Slackia, Olsenella, Desulfovibrio, Butyricimonas,* and *Paraprevotella* (*p* < 0.05 and the logarithm of LDA > 2, Fig. 2b)*.* Furthermore, the first 8 HTC-enriched genera, whose average read counts > 100/sample, were pairwise comparison based on read counts data using FDR-adjusted Wilcoxon rank sum test. In general, read counts of them all showed significant differences among the 6 groups (*Prevotella*: K = 43.659, *p* = 0.000, *Catenibacterium*: K = 39.362, *p* = 0.000, *Clostridium_sensu_stricto*: K = 20.577, *p* = 0.001, *Ruminococcus 2*: K = 21.391, *p* = 0.001, *Faecalibacterium*: K = 15.744, *p* = 0.008, *Holdemanella*: K = 51.247, *p* = 0.000, *Coprococcus*: K = 31.417, *p* = 0.000, and *Parabacteroides*: K = 14.859, *p* = 0.011). The pairwise comparison results showed that *Catenibacterium*, *Clostridium_sensu_stricto*, *Holdemanella*, and *Ruminococcus 2* were enriched in the HTC group than those in the HTN, HHC and LHC groups (*Catenibacterium*: HTC vs. HTN, Z = − 3.024, FDR-adjusted *p* = 0.003; HTC vs. HHC, Z = − 3.605, FDR-adjusted *p* = 0.000; HTC vs. LHC, Z = − 3.558, FDR-adjusted *p* = 0.000. *Clostridium_sensu_stricto*: HTC vs. HTN, Z = − 2.883, FDR-adjusted *p* = 0.018; HTC vs. HHC, Z = − 2.799, FDR-adjusted *p* = 0.018; HTC vs. LHC, Z = − 2.457, FDR-adjusted *p* = 0.029. *Holdemanella*: HTC vs. HTN, Z = − 2.804, FDR-adjusted *p* = 0.007; HTC vs. HHC, Z = − 4.099, FDR-adjusted *p* = 0.000; HTC vs. LHC, Z = − 3.792, FDR-adjusted *p* = 0.000. *Ruminococcus 2*: HTC vs. HTN, Z = − 2.787, FDR-adjusted *p* = 0.009; HTC vs. HHC, Z = − 2.798, FDR-adjusted *p* = 0.009; HTC vs. LHC, Z = − 3.344, FDR-adjusted p = 0.000. Figure 2c). *Prevotella* was enriched in the HTC group compared with HHC and LHC groups (HTC vs. HHC, Z = − 2.529, FDR-adjusted *p* = 0.023; HTC vs. LHC, Z = − 3.103, FDR-adjusted *p* = 0.003, Fig. 2c), but no significant difference was shown between HTC and HTN groups (Z = − 1.474, FDR-adjusted *p* = 0.221). Finally, we confirmed the independent factors associated with CAD by binary logistics regression analysis. First, univariate binary logistic regression analysis was used to compare of clinical characteristics, laboratory characteristics, diet composition and specific bacteria read counts between CAD patients and health individuals. Individuals in the two groups were as the dependent variables (0 = health individual, 1 = CAD). No significant differences were detected in terms of sex, smoking, BMI, DBP, fasting blood glucose, ethnic, living altitude, and fermented, fat, fiber, and protein intake, *Clostridium_sensu_stricto*, *Holdemanella*, *Ruminococcus 2*, and *Prevotella* read counts between the two groups (*p* > 0.05, Table 4 left column). Age, SBP, CRP, carbohydrates intake and *Catenibacterium* read counts were significantly different between the two groups (*p* < 0.05, Table 4 left column). Then, we performed multivariate binary logistic regression analysis to identify the risk factors for CAD. All parameters showing significant differences between CAD and health individual groups in univariate regression analysis were selected for multivariate regression analysis. The results revealed that age (OR = 1.073, 95% CI 1.019–1.130, *p* = 0.007) and SBP (OR = 1.071, 95% CI 1.014–1.132, *p* = 0.014) were independent risk factors for the CAD (Table 4 right column). *Catenibacterium* read counts was also independently associated with CAD (OR = 1.000, 95% CI 1.000–1.001, *p* = 0.021, Table 4 right column). These results showed that compared with altitude- and ethnicity-matched healthy individuals, the composition of the GM in patients with CAD had its own characteristics. The composition of the GM was more unique in CAD patients, especially in the HTC group. *Catenibacterium* might be involved in the development of CAD in Tibetan highlanders.

**Table 4:** Multivariable analysis factors independently related to the presence of CAD.

Variables	Univariate regression		Multivariate regression	
	OR (95%CI)	*p* value	OR (95%CI)	*p* value
Age	1.074 (1.023–1.127)	0.004*	1.073 (1.019–1.130)	0.007*
Sex	0.676(0.300–1.521)	0.344		
Smoking	1.909 (0.855–4.265)	0.115		
BMI	1.196 (0.963–1.484)	0.105		
SBP	1.059 (0.007–1.113)	0.026*	1.071 (1.014–1.132)	0.014*
DBP	0.989 (0.930–1.051)	0.723		
Fasting blood glucose	0.650 (0.496–1.041)	0.073		
CRP	0.717 (0.535–0.961)	0.026*	0.702 (0.511–0.963)	0.028*
Ethnic	0.588 (0.242–1.427)	0.241		
Altitude	1.000 (1.000–1.000)	0.434		
Fermented	0.979 (0.947–1.013)	0.229		
Fat	1.014 (0.986–1.042)	0.336		
Carbohydrates	1.005 (1.000–1.009)	0.036*	1.003 (0.999–1.008)	0.176
Fiber	1.011 (0.985–1.037)	0.411		
Protein	0.990 (0.980–1.001)	0.068		
*Catenibacterium*	1.000 (1.000–1.001)	0.046*	1.000 (1.000–1.001)	0.021*
*Clostridium_sensu_stricto*	1.000 (1.000–1.000)	0.259		
*Ruminococcus 2*	1.000 (1.000–1.000)	0.146		
*Holdemanella*	1.000 (1.000–1.000)	0.569		
*Prevotella*	1.000 (1.000–1.000)	0.162		

Factors with univariate *p* < 0.05 were entered into the multivariate analysis, *p < 0.05.

*OR* odd ratios, *CAD* coronary artery disease, *BMI* body mass index, *SBP* systolic blood pressure, *DBP* diastolic blood pressure, *CRP* c-reactive protein.

### Core network genera

**Figure 3:**
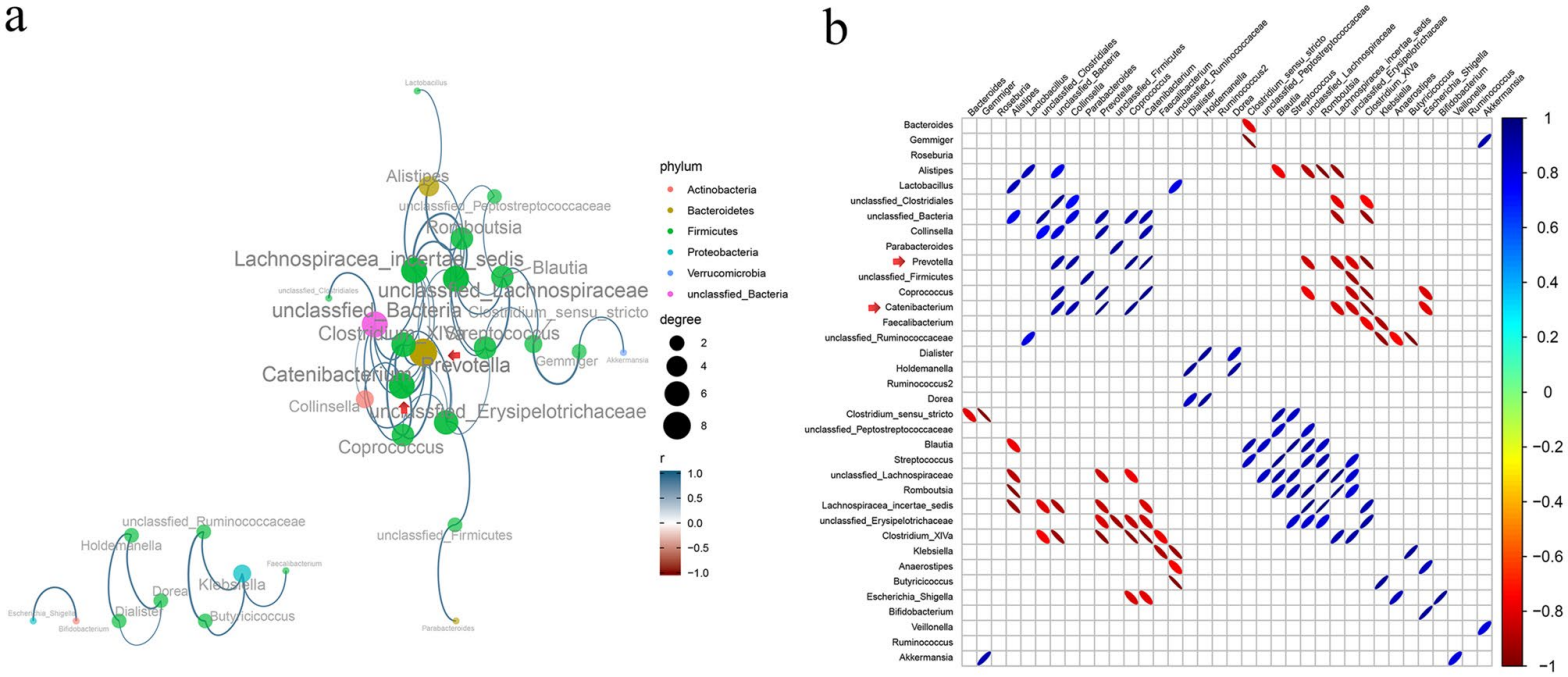
Genus correlation analysis. (**a**) Genus correlation network (“igraph” package in R sofware v.3.6.0, https://www.r-project.org/). Different color of the circles represent different phylum of different genus. Size of the circles and color of the lines represent the degree of correlation. Size of the circles change with the right size scale and color changes with the color scale at the bottom right. (**b**) Corrplot of genus correlation (“corrplot” package in R sofware v.3.6.0, https://www.r-project.org/). Size of the ellipses in the diagram represent the absolute value of the correlation coefficient (the greater the absolute value of the correlation, the smaller the ellipse), the right-slanted ones are positive correlation, the left-slanted ones are negative correlation, and the color changes with the right color scale. The figure only shows correlated genus when statistically significant, *p < 0.05 and FDR < 0.05.

Microbial interactions cannot be ignored in maintaining microbial homeostasis, so the network among the genera was investigated. *Prevotella* was positively correlated with *Catenibacterium* (r = 0.478, FDR-adjusted *p* = 0.000), *Coprococcus* (r = 0.392, FDR-adjusted *p* = 0.000), and *Collinsella* (r = 0.344, FDR-adjusted *p* = 0.010), but negatively correlated with *Clostridium_XIVa* (r = − 0.192, FDR-adjusted *p* = 0.000) and *Lachnospiracea_incertae_sedis* (r = − 0.445, FDR-adjusted *p* = 0.020) (Fig. 3A,B)*.* Similarly, *Catenibacterium* was positively correlated with *Prevotella* (r = 0.478, FDR-adjusted *p* = 0.000), *Coprococcus* (r = 0.218, FDR-adjusted *p* = 0.010), and *Collinsella* (r = 0.364, FDR-adjusted *p* = 0.020), but negatively correlated with *Clostridium_XIVa* (r = − 0.158, FDR-adjusted *p* = 0.010) and *Lachnospiracea_incertae_sedis* (r = − 0.259, FDR-adjusted *p* = 0.030) (Fig. 3A,B)*.* In conclusion, the genus network results indicated that *Prevotella* and *Catenibacterium* were the core genera in the network diagram.

### Correlation between specific bacteria, general characteristics, and diet composition

**Figure 4:**
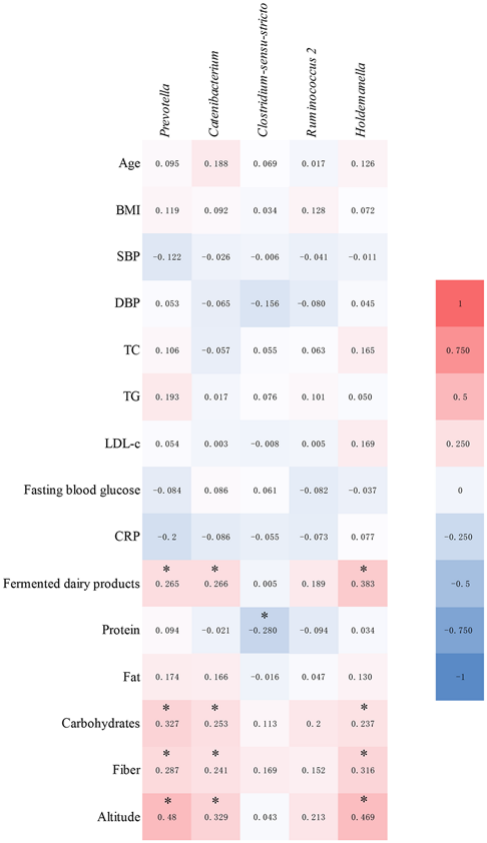
Spearman’s correlation matrix heatmap between general characteristic, diet composition, and specific bacteria. the color represents positive (red) or negative (green) correlations, the color changes with the right color scale, and *p < 0.05 and FDR < 0.05. *BMI* Body Mass Index, *SBP* systolic blood pressure, *DBP* diastolic blood pressure, *TC* total cholesterol, *TG* triglyceride, *LDL-c* low-density lipoprotein cholesterol, CRP c-reactive protein.

Next, the link between HTC-specific bacteria, general characteristics, and diet composition were estimated by Spearman’s correlation analysis. The results showed that *Catenibacterium* was positively correlated with altitude (r = 0.329, FDR-adjusted *p* = 0.000), fermented dairy product (r = 0.266, FDR-adjusted *p* = 0.008), carbohydrate (r = 0.253, FDR-adjusted *p* = 0.015), and fiber (r = 0.241, FDR-adjusted *p* = 0.015) intake by subjects (Fig. 4). Similarly, *Holdemanella* was positively correlated with altitude (r = 0.469, FDR-adjusted *p* = 0.000), fermented dairy product (r = 0.383, FDR-adjusted *p* = 0.000), carbohydrate (r = 0.237, FDR-adjusted *p* = 0.019), and fiber (r = 0.316, FDR-adjusted *p* = 0.000) intake by subjects (Fig. 4). *Prevotella* was also positively correlated with altitude (r = 0.480, FDR-adjusted *p* = 0.000), fermented dairy product (r = 0.265, FDR-adjusted *p* = 0.008), carbohydrate (r = 0.327, FDR-adjusted *p* = 0.000), and fiber (r = 0.287, FDR-adjusted *p* = 0.005) intake by subjects (Fig. 4). Whereas, *Clostridium_sensu_stricto* was negatively correlated with protein (r = − 0.280, FDR-adjusted *p* = 0.015) intake by subjects (Fig. 4). These results indicated that enriched genera *Catenibacterium*, *Holdemanella*, and *Prevotella* in HTC might correlated with high fermented dairy product, carbohydrate, and fiber intake by them, and enriched genera *Clostridium_sensu_stricto* in HTC might correlated with low protein intake by them.

## Discussion

CAD is a disease affected by multiple factors, and exploring the pathogenesis of CAD is still the goal of many researchers. Recently, studies have shown that the GM and its metabolite, TMAO, are closely related to CAD^14,15,18^. GM disorder is considered one of the potential factors involved in the pathogenesis of CAD. Because of the unique genetic background, living environmental factors and diet of Tibetan Highlanders, the incidence and mortality of individuals in these populations suffering from CAD are different from those of other populations^4–6^. Interestingly, the incidence of CAD in Tibetan Highlanders is lower than that in Japanese individuals living at sea level, which suggests that unknown contributors play protective roles in the CAD of Tibetan Highlanders.

Disorders of the GM may lead to the occurrence of diseases, but the optimization of the GM may prompt the body to adapt to the environment. Several studies have shown that Tibetan Highlanders showed different GM characteristic when compared with other individuals, which might be because stable and balanced gut ecosystems play an important role in human self-protection in harsher environments^16,19,20^. In this study, our results showed that α-diversity, measured as microbial richness and evenness by the Shannon, Ace, and Chao indexes, were higher in the HTN group than in the HHN and LHN groups. And β-diversity, measured as microbial distribution by weighted UniFrac distance matrix-based PCoA, was different in the HTN group compared with it in the HHN and LHN groups. There results are in accordance with what Liu et al. reported^21^. Moreover, we also found that Shannon, Ace, and Chao indexes were higher in the HTC group than in the HHC and LHC groups. And weighted UniFrac distance matrix-based PCoA in the HTC group formed different cluster comparing with those in the HHC and LHC groups. There results indicated that host genetic and environmental factors shaped the diversity and distribution of GM in both healthy individuals and patients suffered from CAD. Moreover, this might be one of the reasons why CAD was not prevalent in Tibetan Highlanders.

To date, data suggests that both *Prevotella* and *Catenibacterium* are closely correlated with dietary habits, living environments and/or ethnicities. Wu et al.^22^ reported that enterotypes dominated by *Prevotella* and *Catenibacterium* were strongly associated with long-term diets containing high carbohydrates but little protein and animal fat. Several studies have demonstrated that *Prevotella* is one of the core microbiota genera of the Tibetan population, and its high abundance is associated with high carbohydrate and low fat and protein intake^16,23,24^. Dehingia et al.^25^ confirmed that the abundance of *Prevotella* was positively correlated with the industrialization level of the living environment. He et al.^26^ reported that supplying a diet with additional oat bran, which is a food containing high fiber, increased the abundance of *Prevotella* and *Catenibacterium* in the gut of the pigs. *Catenibacterium* also correlated with ethnicity. A higher abundance of *Catenibacterium* was noted in Indian adults than in Chinese adults^27^. *Catenibacterium* was also found to be enriched in the gut of Egyptian children and Bangladeshi children compared to US children^28,29^. By analyzing the diet structure of our subjects, we also confirmed that Tibetan Highlanders consumed more carbohydrates, fiber and fermented dairy products. The industrialization level of the QTP is much lower than that of Wuhan, which is a large modern city. Moreover, both genera *Prevotella* and *Catenibacterium* were enriched in Tibetan Highlanders compared with healthy condition-matched Han individuals (HTC vs. HHC and LHC; HTN vs. HHN and LHN). In addition, *Prevotella* and *Catenibacteriumn* were closely positively correlated with each other. Furthermore, we also confirmed that *Prevotella* and *Catenibacterium* were positively correlated with altitude, and fermented dairy product, carbohydrate and fiber intake by the subjects. Therefore, our study confirmed that enriched *Prevotella* and *Catenibacterium* in gut of Tibetan Highlanders probably associated with unique dietary habits and living environments of this population.

More importantly, we found that the abundances of *Catenibacterium* was higher in the HTC group than in the HTN group, which demonstrated that *Catenibacterium* is related to the progression of CAD. Accumulating evidence indicates that *Catenibacterium* might play protective roles in cardiovascular diseases. It is known that some carbohydrates and dietary fiber can escape digestion from upper gastrointestinal tract and are fermented by GM in the caecum and colon^30^. The most abundant metabolites produced after carbohydrates and fiber are broken down by GM are SCFAs, which exerted beneficial effects on regulation of inflammation and slowing down the development of atherosclerosis^31–33^. One study reported that *Catenibacterium* could improve gut health and nutrient utilization by enhancing the fermentation of fiber to produce SCFAs^26^. Fu et al.^34^ reported that *Catenibacterium* was inversely correlated with host BMI. Kelly et al.^35^ showed that enrichment of *Catenibacterium* was associated with a decreased lifetime CAD risk. Therefore, it can be assumed that *Catenibacterium* is special genera induced by the unique dietary habits of Tibetan Highlanders and might contribute to Tibetan Highlanders suffered from CAD.

*Clostridium_sensu_stricto* is another fermentative bacteria and beneficial for host by producing SCFAs^36^*,* but the role of *Clostridium_sensu_stricto* in CAD is till unclear. Fan et al.^37^ confirmed that fed pig with low level protein diet, the proportion of *Clostridium_sensu_stricto* in colon was decreased. Here we found that *Clostridium_sensu_stricto* was enriched in the HTC group than it in the HTN group, as well as than it in HHC and LHC groups. Moreover, enriched *Clostridium_sensu_stricto* was negatively correlated with protein intake by subjects. So, *Clostridium_sensu_stricto* might be another potential essential bacteria associated with HTC.

Gut ecosystem linked with variable factors, including general characteristics (like age, sex, BMI, et al.), health status, dietary habit, ethnicity, geography, altitude and civilization. Tibetans are unique ethnicity with tough living environments and different dietary habits. Studies have demonstrated GM is involved in the progression of CAD, but little is known about the specific feature of GM in different ethnic groups from different geographical locations who suffering from CAD. In this study, we demonstrated that the GM of Tibetan Highlanders suffering from CAD showed higher α-diversity and a distinct cluster compared with healthy Tibetan Highlanders and Han CAD patients living at high and low altitudes. The beneficial genera *Catenibacterium* and *Clostridium_sensu_stricto* were enriched in Tibetan Highlanders, suffering from CAD compared with healthy Tibetan Highlanders and Han CAD patients living at high or low altitude. Moreover, *Prevotella* and *Catenibacterium* positively correlated with each other and were core genera in the genus co-network. Additionally, *Catenibacterium*, *Holdemanella*, and *Prevotella* were positively correlated with fermented dairy product, carbohydrate and fiber intake by the subjects, while *Clostridium_sensu_stricto* was negatively correlated with protein intake by subjects. In conclusion, our study indicated that Tibetan Highlanders suffering from CAD showed distinguishing GM, which was linked to their unique dietary characteristic and might associated with CAD. However, our study still have some limitations. First, the sample size of the HTC group is small. There are two reasons for this limitation. On one hand, CAD inclusion standards were very strict in this study. CAD is a disease affected by multiple factors. Hypertension, diabetes, and obesity are major risk factors of CAD. However, many current studies have reported that the GM dysbiosis was found correlated with the development of hypertension, diabetes and/or obesity^38–40^. Moreover, there are several types of CAD, the status of GM might also be different in these sub-types. One study has confirmed there were no significant differences in the diversity of the GM between the healthy control subjects and the patients with stable angina^10^. In the acute phase, patients with myocardial infarction often passively change their diet, daily life, and defecation habits, while these factors closely link to diversity of GM. In order to avoid the confounding factors caused by these comorbidities and different types of CAD, all CAD patients recruited in this study were unstable angina (UA), and all UA those who had comorbidities, including hypertension, diabetes, obesity, heart failure, renal failure, stroke, peripheral artery diseases, or any other acute or chronic inflammatory diseases were excluded. On the other hand, Tibetan Highlanders having a lower incidence of CAD than plain-living individuals. So, it takes a long time for a single center to raise a large number of HTCs. Second, medication is a major confound factor in this study. Ideally, the comparison should be between CAD and healthy control before medication. But from an ethical point of view, we must give medication to patients diagnosed with CAD. Actually, in several such studies, CAD patients were medicated. In the research finished by Jie et al.^41^ patients with CAD used several medicines, including acarbose and atorvastatin. They proved that CAD statue, but not those drug used, caused the major distinguishing feature of GM in CAD patients. In the study conducted by Emoto et al., medication also did not matched between CAD and control groups^42^. In this study, we recruited CAD patients were administered aspirin, statins, angiotensin-converting enzyme inhibitor/angiotensin II receptor blocker and β blocker, which are all essential drugs for secondary prevention of CAD. Therefore, we assume that these medications might weaken the disease signal, which meanings an even more significant difference would be expected if the study was free of medication. Due to these limitations, multi-center, larger-scale, drug-free studies are needed to verify our results. As HTC-specific genera, *Catenibacterium* and *Clostridium_sensu_stricto*, are both SCFAs producing bacterial. Next, we will study whether *Catenibacterium* and *Clostridium_sensu_stricto* are involved in Tibetan Highland CAD development by producing SCFAs and mediating inflammatory response in a mouse model of atherosclerosis that simulates Tibetan living environment and dietary habits.

## Methods

### Ethics statement

All experimental protocols were established according to the ethical guidelines of the Helsinki Declaration and approved by Ethics Committee of the Qinghai University, Xining, China (Ethical approval number: P-SL-2017022 and P-SL-2017063). The study obtained written informed consent from all participants.

### Subjects

We recruited a total of 36 patients with CAD and 104 healthy volunteers from May 30, 2018 to May 30, 2019. All CAD patients in this study were UA. UA is defined as a normal measurement of cardiac troponin and has at least one of the following criteria: prolonged (> 20 min) resting angina, new-onset angina, crescendo angina or post-myocardial infarction angina^43^. All healthy volunteers were physically and psychologically healthy. Both CAD patients and healthy volunteers were divided into three groups: 8 CAD patients and 34 healthy individuals were Tibetans living at QTP (altitude: 2260–4500 m, HTC and HTN, respectively). 14 CAD patients and 35 healthy individuals were Han individuals residing at QTP for at least 10 years (altitude: 2000–3100 m, HHC and HHN, respectively). Another 14 CAD patients and 35 healthy individuals were Han individuals living in Wuhan, which is a large modern sea level city in southern China (altitude: 13 m, LHC and LHN, respectively). All CAD participants were administered aspirin, statins, angiotensin-converting enzyme inhibitor/angiotensin II receptor blocker and β blocker. None of the participants had hypertension, diabetes, obesity, cancer, gastrointestinal diseases, heart failure, renal failure, stroke, peripheral artery diseases, or any other acute or chronic inflammatory diseases. None used any antibiotics or probiotics within the past 3 months. None was pregnant or lactating. None of them used any other drugs, such as metformin, insulin, or traditional Chinese medicines, et al. The defecation habit of all participants was 1–2 times/day.

### Stool sampling

Approximately 200 mg fresh stool samples in the morning from all individuals were collected in 10 mL sterile tubes without RNAse, and were immediately placed on ice and transferred into a − 80 °C deep freezer for cryopreservation until used for DNA extraction.

### DNA extraction

DNA was extracted from fecal samples using the E.Z.N.A. Soil DNA Kit (M5635-02, Omega, USA), following the manufacturer’s instructions. The concentration and quality of DNA were determined using a Qubit 3.0 fluorometer (Q10212, Life Technologies, USA).

### 16S rRNA gene amplification

The V3–V4 (341F-805R) region of 16S rRNA was amplified by PCR using Taq Master Mix (2×) (P111-03, Vazyme, China). The PCR primers sequences were F: CCTACGGGNGGCWGCAG and R: GACTACHVGGGTATCTAATCC. Unique barcodes were tagged to the 5′-end of the 341F primer to split the sequences of each sample. PCR amplification was performed in duplicate. The PCR reaction mix was prepared as follows: 2 × Taq master Mix (15 μL), PCR primer F and primer R (10 μM, 1 μL, respectively), and template DNA (10 ng). The reaction volume was brought up to 30 μl with ddH2O. PCR was carried out using T100^TM^ Thermal Cycler (BIO-RAD, USA) and the conditions were 3 min at 94 °C followed by 5 cycles of 94 °C for 30 s, 45 °C for 20 s, and 65 °C for 30 s, then 20 cycles of 94 °C for 20 s, 55 °C for 20 s and 72 °C for 30 s, and finally 5 min at 72 °C. PCR products were purified using Agencourt AMPure XP Kit (Beckman Coulter, USA) and were sequenced using Illumina Miseq^TM^ (Truseq Series, Illumina, USA) by Sangon Biotech Co. Ltd., Shanghai, China.

### Bioinformatics analysis

The original image data files obtained by Illumina Miseq™ were converted into Raw Reads by Base Calling analysis. First, the primer adapter sequences were removed, and then the paired-end reads were merged into single sequences, and then the sequences were identified and distinguished by the barcodes, and finally quality control filtering was performed to obtain valid data for each sample. All high-quality sequences were clustered into OTUs at a 97% sequence similarity, and the OTU table was generated using USEARCH for OTU cluster analysis. The Shannon index, Ace index, and Chao index and rarefaction curves were calculated by mothur software (v.1.43.0)^44^ and plotted in the R studio. The weighted UniFrac distance matrices were calculated using mothur and visualised by PCoA within the R (v.3.6.0), and statistical significance of bacterial distribution was evaluated by the adonis in R. The barplot was drawn using circlize package in R. LEfSe was drawn using the LEfSe software (v.1.1.0)^45^. The genera enrichment of each group was compared at read counts levels, and the box plot was drawn using SPSS (version 26). The correlation network and corrplot diagram were used to show link between every two genus. The correlation coefficient were calculated with SparCC and the diagram were drawn using igraph package (v.1.0.1) and corrplot package in the R studio, respectively. Bacterial abundance above 1% was analyzed. The the correlation matrix heat map was illustrated by Excel (v.2013) and the correlation coefficient were calculated by SPSS.

### Statistical analysis

Data was analyzed by SPSS (v.26) and R software (v.3.6.0). The Shapiro–Wilk test was used to determine whether or not the measurement data was normally distributed. For normally distributed measurement data, one-way ANOVA and the two-tailed Student’s *t*-test were used to test differences among more than two groups and between two groups, respectively. For non-normally distributed measurement data and categorical variables, Kruskal–Wallis test was used to test the significance of differences among different groups. For pairwise comparison read counts of genera, Wilcoxon rank sum test was used. To discover the link between two parameters, Spearman’s correlation analysis was used, and Spearman’s correlation coefficient was calculated. Levels of genera *Prevotella*, *Catenibacterium*, and *Escherichia_Shigella* were adjusted for traditional CAD risk factors (including age, sex, SBP, DBP, fasting blood glucose, BMI, smoking status, and CRP), ethnicity, and altitude by quadratic logistic regression analysis. For all tests, a value of *p* < 0.05 was considered statistically significant. For multiple comparison, p-value was adjusted by FDR at a threshold of < 0.05 or < 0.08 was considered for statistical significant. For LEfSe analysis, *p* < 0.05 and the logarithm of LDA > 2 was considered for statistical significant.

### Accession numbers

The rawdata of 16 s RNA from the present study have been uploaded to the SRA database under the accession number SUB9029131.
